# Application of a Hybrid 3D-2D Laser Scanning System to the Characterization of Slate Slabs

**DOI:** 10.3390/s100605949

**Published:** 2010-06-14

**Authors:** Marcos López, Javier Martínez, José María Matías, José Antonio Vilán, Javier Taboada

**Affiliations:** 1 Department of Mechanical Engineering, University of Vigo, 36310 Vigo, Spain; E-Mail: jvilan@uvigo.es (J.A.V.); 2 Department of Environmental Engineering, University of Vigo, 36310 Vigo, Spain; E-Mails: javier.martinez@uvigo.es (J.M.); jtaboada@uvigo.es (J.T.); 3 Department of Statistics, University of Vigo, 36310 Vigo, Spain; E-Mail: jmmatias@uvigo.es (J.M.M.)

**Keywords:** laser scanner, slate slabs, tsai calibration, digital camera

## Abstract

Dimensional control based on 3D laser scanning techniques is widely used in practice. We describe the application of a hybrid 3D-2D laser scanning system to the characterization of slate slabs with structural defects that are difficult for the human eye to characterize objectively. Our study is based on automating the process using a 3D laser scanner and a 2D camera. Our results demonstrate that the application of this hybrid system optimally characterizes slate slabs in terms of the defects described by the Spanish UNE-EN 12326-1 standard.

## 1. Introduction

The control of the quality of slate slabs, from the initial slate block cutting tasks to the point when suitably sized slabs are obtained by a stonemason, continues to be a manual process.

The process of obtaining slates to be used for roofing commences with the extraction of slate blocks from the quarry. These blocks are transported to the processing plant in dump trucks and unloaded onto a bed of earth. If a mechanical cutting system is used, the blocks are unloaded using a small crane so as to minimize damage and take advantage of existing cuts. Blocks are exfoliated along the schistosity planes using a jackhammer with a flat bit. A hammer and stonecutter’s chisel are used to obtain slate slabs, cut according to the schistosity planes. Finally, slate slabs are cut to commercial sizes using a guillotine or pedal-operated cutters, with the operator choosing the final size according to the cut allowed by the slate thin section.

Each slate slab undergoes quality control to detect possible defects before being assigned to a particular use. Quality control, performed manually by an expert, has the drawback that it is subjective and aggravated by the possible effects of fatigue. Furthermore, experts need specific and costly training. There is, therefore, a need to develop a more reliable automated passive (non-intrusive) system. In its product specification section, the Spanish UNE-EN12326-1 standard [1] defines the defects that characterize roofing slate. Roofing slate quality, furthermore, has been the subject of a number of studies [2,3]. The defects analysed in this study are described as follows:

- **Material defects:** Slate slabs, divided into thirds, need to be checked for material defects in each third. The ratio (%) between the slab surface area and the theoretical ideal rectangular contour area contained in the slab was calculated.
- **False squaring:** Slate slabs need to be as rectangular in shape as possible. The degree of difference between each angle and a right angle is analysed.
- **Warping:** Warping in slates may result in breakage when slate is being fitted. This is expressed as the difference in millimetres between the height of a corner compared to the rest of the slate.
- **Fissuring:** Cracks are assessed by calculating the mean surface area and studying the standard deviation of the typical distribution in order to evaluate homogeneity. The standard does not describe limit values for this criterion (as it tends to depend on commercial varieties); in this case, the limit value based on expert opinion given in Table 1 was used [2].
- **White (flowerlike) staining:** Flowerlike stains have tones that are clearly different from the rest of the slate. Studied, therefore, is the area of the stains identified in the image.

**Figure 1.**
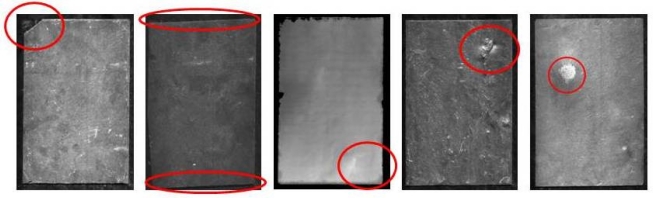
Defects identified. Left to right: material defects, false squaring, warping, fissuring and white staining.

Figure 1 illustrates the defects analysed in this study and Table 1 indicates the limit values established for this particular application, based on the values given in the ruling standard. The above list covers all the defects referred to in the UNE standard, with the exception of quartz filaments, which will be dealt with in future research.

Bearing in mind the information extracted from the UNE standard, the most restrictive slab slate size for all the defects listed is estimated to be 0.5 mm length- and crossways and 0.1 mm in terms of thickness.

This study was based on artificial vision techniques, given that they are non-intrusive. We chose the hybrid 2D-3D laser scanner technology over other techniques (such as stereo vision) because it is more robust [4]—a key aspect given the industrial setting of the application. Furthermore, commercial artificial vision systems produce very accurate results for our kind of problem.

Precision results basically from the resolution of the camera sensors, which, in turn, depends on the field of view (FOV) necessary. In our particular case, the FOV was 340 × 240 mm length- and crossways, resulting in a minimum 2D camera resolution of 960 pixels. As for thickness (affecting the 3D scanner), the FOV was 4 mm, requiring a minimum resolution of 80 pixels. Different measurement techniques have been used to inspect ceramic slabs for quality (e.g., [5]). There are four main approaches to characterization:

- *Methods based on differentiating between grey tones* [6], with small differences in the grey scale reflecting defects in the slate.
- *Morphological methods* [7], based on assessing the shape and surface of the defects [8].
- *Local binary pattern methods* [9], based on the construction of an invariant texture measure based on the grey scale and derived from a general definition of texture in a local environment.
- *Texture analysis* [10–11], in which texture is the criterion used to differentiate between slate qualities.

Since texture is a key feature of slate, Ghita [12] proposed using a 2D viewing system inspired by different studies in which a diffuse lighting method was used [13,14]. We studied the feasibility of including, as part of the slate classification process, a 3D laser scanning system that would make geometric measurements of the slate slabs in three dimensions (see [15–18] for engineering applications to industrial inspection processes). However, to overcome the problem of a costly prior calibration process, we developed our own simple calibration algorithm as a particular case of the Tsai method [19]. This simplified approach substantially reduced the complexity of the calibration. The article is structured as follows: firstly, the methodology is described in terms of the equipment and the calibration algorithm used; secondly, the results obtained using this methodology are described, focusing on those of most significance; and finally, the most relevant conclusions arising from our research are discussed.

## 2. Methodology

### 2.1. Equipment

For our proposed 3D-2D hybrid control system, in a first inspection phase, different kinds of slate defects (described in more detail below) were identified using dimensional control implemented in 3D. In a second phase, white (flowerlike) staining of the slate surface were identified using the 2D system.

The cameras used for this artificial vision system were the following:

- For 3D characterization, a Ranger C55 camera, with a resolution of 1,546 × 512 and a speed of 30,000 profiles per second.
- For 2D characterization, a DALSA Spyder3 linear camera, with a resolution of 2,048 pixels and a speed of up to 18,000 lines per second.

The system was configured so that each slate slab would be fully included in the images created by both the 2D and 3D systems, with the FOV greater than the slab dimensions. Furthermore, the 3D laser camera viewing field (and the laser angle) was established in such a way that it could account for surface defects up to 7.5 mm over the mean height of the slate slabs.

**Figure 2.**
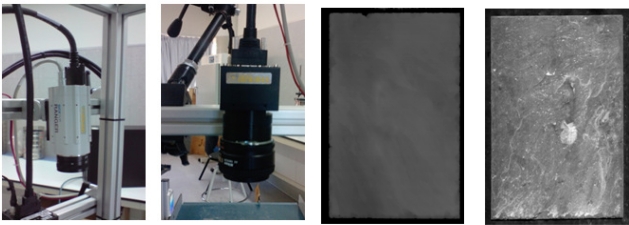
The 3D laser scanner and the 2D linear camera (left) with sample images for each (right).

Figure 2 shows the 3D laser scanner (left) and the 2D linear camera (right) used for this study, along with a sample image for each. Lighting as a particularly important part of an artificial vision system was selected according to the imaging process:

- A laser unit for the PowerLaser model for 3D.
- A LED HLND red light unit for auxiliary illumination for the 2D linear camera.

**Figure 3.**
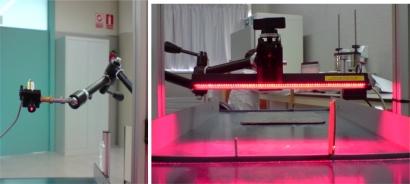
Lighting systems used: A laser unit for 3D (left) and red light unit for 2D (right).

Figure 3 shows the lighting systems used for this study. To ensure that the processes did not interfere with each other, an order blocking filter (OBF) was used in the 3D sensor to cut specific wavelengths and so improve the robustness of the 3D system.

**Figure 4.**
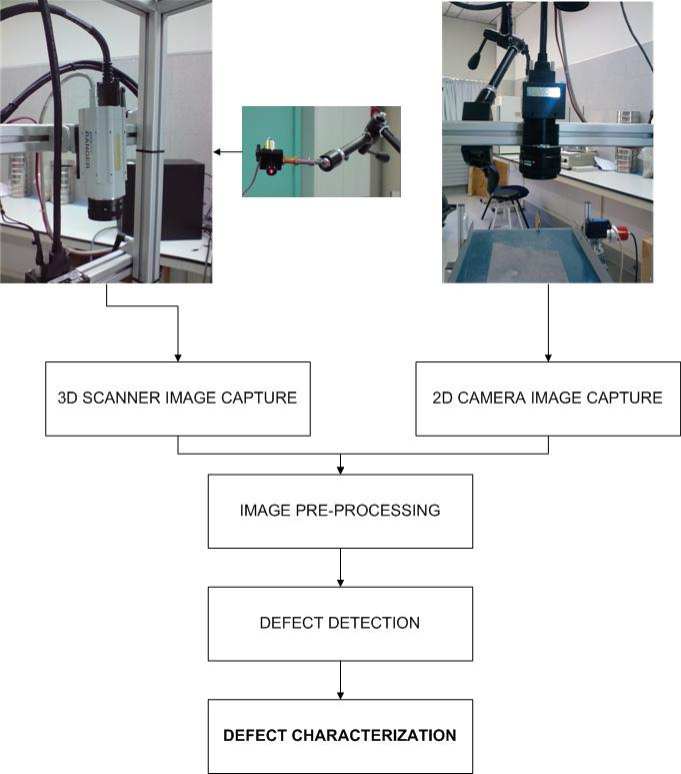
Flow chart illustrating the hybrid characterization system for slate slabs.

A flow chart illustrating the hybrid characterization system for slate slabs is depicted in Figure 4.

**Figure 5.**
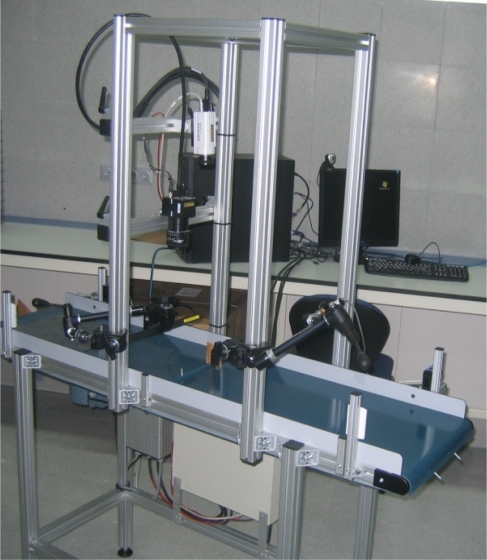
Prototype automated slate slab classification system.

In terms of automating the entire process, a prototype was built that included a conveyor belt, 3D camera, 2D linear camera and lighting systems (Figure 5). The model was implemented in real time so as to be adaptable to the requirements of a future industrial application.

Images are captured in response to a command issued by a trigger that detects the passing of the slate. The conveyor belt is configured for a maximum speed of 30 metres/minute, and acceleration and deceleration ramps are also configurable so as to make the prototype as similar as possible to a future industrial application. The application is not affected by changes in speed thanks to an encoder, installed in the motorized conveyor belt roller, responsible for varying electric pulse frequency according to the speed of the belt.

**Figure 6.**
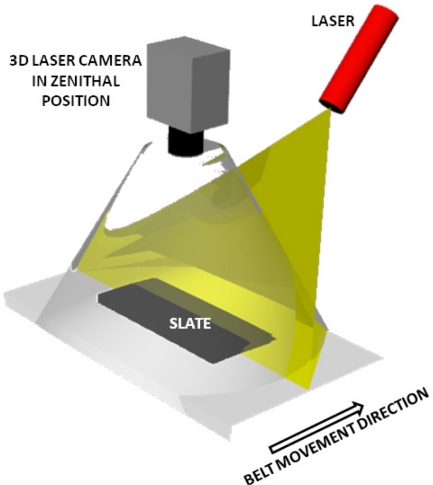
Diagram of the 3D sensing system.

Figure 6 shows a diagram of the 3D sensing system, with the camera in the zenithal position, the laser plane requiring calibration, the direction of the conveyor belt and the slate slab in position on the belt.

### 2.2. Calibration Algorithm

**Figure 7.**
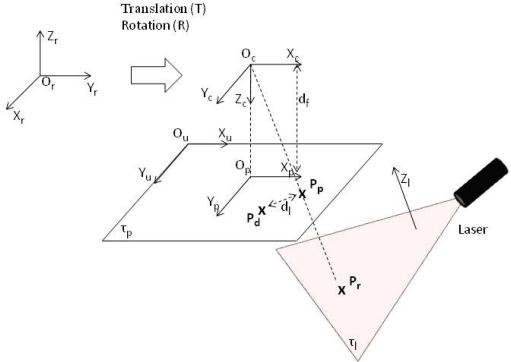
Diagram representing camera and world coordinates for laser plane calibration in the general case.

The camera calibration method described by Tsai [19] is the most frequently used for this kind of application. Because this system includes a 3D laser scanner, co-planar calibration is necessary and, consequently, the radial alignment proposed by Zexiao [20] is taken into account. Figure 7 depicts the camera and world coordinates relevant to the Tsai calibration process.

Let *O_r_X_r_Y_r_Z_r_*, *O_c_X_c_Y_c_Z_c_*, *O_p_X_p_Y_p_Z_p_*, *O_u_X_u_Y_u_* be the reference systems for the real world, the camera (after translation **T** and rotation **R**), the projection plane ***τ***_*p*_ (where *d_f_* is the effective focal distance) and the sensor, respectively.

Let *P_r_* = (*x_r_*, *y_r_*, *z_r_*) be a point in the real coordinates; projection of this point on the projection plane corresponds to *P_p_* = (*x_p_*, *y_p_*), but, bearing in mind lens distortion, the projection corresponds to *P_d_* = (*x_d_*, *y_d_*). Furthermore, let (*x_u_*, *y_u_*) be the coordinates for a point in the projection plane and *x_uo_p__*, *y_uo_p__* the coordinates for the point *O_p_* with respect to the system *O_u_X_u_Y_u_* expressed in pixels.

Denoting the rotation matrix **R** = (*r*)*_ij_* and translation matrix **T** = (*T_x_*,*T_y_*,*T_z_*) that intervene in the transformation of the reference system *O_c_X_c_Y_c_Z_c_*, we obtain the following calibration equations:

(1) $$\frac{\left(x_{u}-x_{{\mathrm{uo}}_{p}}\right)}{N_{x}}(1+k_{1}\;q^{2})=d_{f}\;\frac{r_{11}\;x_{r}+r_{12}\;y_{r}+r_{13}\;z_{r}+T_{x}}{r_{31}\;x_{r}+r_{32}\;y_{r}+r_{33}\;z_{r}+T_{z}}$$

(2) $$\frac{\left(y_{u}-y_{{\mathrm{uo}}_{p}}\right)}{N_{y}}(1+k_{1}\;q^{2})=d_{f}\;\frac{r_{21}\;x_{r}+r_{22}\;y_{r}+r_{23}\;z_{r}+T_{y}}{r_{31}\;x_{r}+r_{32}\;y_{r}+r_{33}\;z_{r}+T_{z}}$$

with $q^{2}={\left(\frac{\left(x_{u}-x_{{\mathrm{uo}}_{p}}\right)}{N_{x}}\right)}^{2}+{\left(\frac{\left(y_{u}-y_{{\mathrm{uo}}_{p}}\right)}{N_{y}}\right)}^{2}$, where *N_x_* and *N_y_* are the number of pixels per unit of length in the digital image along the axes *X_u_* and *Y_u_*, respectively, and where *k*_1_ is the first-order radial distortion coefficient for the lens.

As with the camera and its projection plane, it is also necessary to calibrate the laser triangulation plane ***τ***_1_ [21]. Let *A^l^x_c_* + *B^l^y_c_* + *C^l^z_c_* + *D^l^* = 0 be the hyperplane equation referring to the system of coordinates *O_c_X_c_Y_c_Z_c_*. The following must be added to the calibration equations (1)–(2):

(3) $$\begin{array}{l} A^{l}(r_{11}\;x_{r}+r_{12}\;y_{r}+r_{13}\;z_{r}+T_{x})+B^{l}(r_{21}\;x_{r}+r_{22}\;y_{r}+r_{23}\;z_{r}+T_{y})+\\ +C^{l}(r_{31}\;x_{r}+r_{32}\;y_{r}+r_{33}\;z_{r}+T_{z})+D^{l}=0 \end{array}$$

Hence, using the indicated notation, the system (1)-(2)-(3) corresponds to the system of Tsai calibration equations and the laser plane.

The following aspects need to be taken into account in the calibration process:

- Measurement is made on a flat conveyor belt. However, even though the belt may have been precision-manufactured and moves over an absolutely flat surface, it is not possible to calibrate all the points of the belt, as the system occasionally stops for calibrations or other reasons while the belt keeps moving. Consequently it is not always possible to know which section of the belt is being scanned.
- Although it is necessary to estimate the dimensions and texture features of the slate slab, the measurements do not need to be absolutely precise. In other words, a measurement error margin of 5% is admissible, given that the goal is inspection/detection.
- Speed is important for slate characterization systems (the greatest number of slate measurements in the least possible time) as production line speed (30 metres/minute) is constant.

Bearing in mind these issues, we applied the following hypotheses to the Tsai calibration model so as to speed up processing time and reduce the number of variables:

1. The camera is in exact zenithal position and the sensor is also in exact zenithal position to ensure parallelism with the measurement plane; that is, **R** = **I**.
2. There is no radial distortion, hence, *k*_1_ = 0.
3. There is no difference between resolutions along different directions, hence, *N_x_* = *N_y_*.
4. The laser beam passes through the *Y_c_* axis, thus, *A^l^x_r_* + *C^l^z_r_* = 0.
5. Slabs have a small thickness relative to movement, hence, *z_r_* ⋘ *T_z_* ⇒ *z_r_* + *T_z_* ≃ *T_z_*.

Bearing in mind the hypotheses in (1)-(2)-(3), the calibration problem is written as:

(4) $$\frac{\left(x_{u}-x_{{\mathrm{uo}}_{p}}\right)}{N_{x}}=d_{f}\;\frac{x_{r}+T_{x}}{z_{r}+T_{z}}≃d_{f}\;\frac{x_{r}+T_{x}}{T_{z}}$$

(5) $$\frac{\left(y_{u}-y_{{\mathrm{uo}}_{p}}\right)}{N_{x}}=d_{f}\;\frac{y_{r}+T_{y}}{z_{r}+T_{z}}≃d_{f}\;\frac{y_{r}+T_{y}}{T_{z}}$$

(6) $$A^{l}x_{r}+C^{l}z_{r}=0$$

These hypotheses are entirely valid for the case of slate, as the loss of precision is offset by the simplicity of the process. Furthermore, for our industrial application, a small loss (of around 5%) in precision is acceptable. It should be borne in mind that calibration should be repeated periodically, given the nature of the slate processing industry (dirt, vibrations, *etc.*). Note that the calibration algorithm in (4)-(5)-(6) considerably reduces not only the cost of operations, but also the complexity, making it possible to use less expert staff.

## 3. Application and Results

### 3.1. Application

In order to study the application of the vision system to the characterization of slate, we used a sample of 75 slate slabs measuring approximately 320 × 220 mm. Only 10% of the slabs had none of the defects described above. This was so that the algorithm could be implemented with the largest possible number of defective slabs.

To detect the defects described above, we used several preprocessing procedures and artificial vision applications. For the Region of Interest (ROI):

- Preprocessing: Algorithms implemented in a phase preliminary to the execution of the program,
  ○ *Threshold*. Binary filter where 1 and 0 are assigned to pixels above and below, respectively, a certain value.
  ○ *Expand*. Associates the pixel value with its neighbourhood.
  ○ *Shrink*. Eliminates pixels that can be rated as noise.

**Figure 8.**
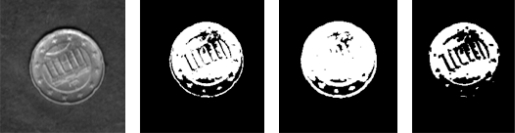
Results for the different pre-processes used. Left to right: the real image, the threshold result, the expand result and the shrink result.

Figure 8 depicts an example of the different pre-processes used for the case of a coin placed on a slab. The image on the extreme left shows a real depiction of the coin, and the three remaining images, left to right, show the results for the threshold algorithm, the expand algorithm (taking into account neighbourhood values) and the shrink algorithm (ruling out possible noise).

- Processing:
  ○ *Connectivity*. Identifies binary large objects (BLOB) by seeking associations between pixels with the same value in different neighbourhood directions.
  ○ *Statistics*. Conducts a routine statistical analysis (mean, mode, median, standard deviation, *etc.*) of the values of the pixels in the ROI.
  ○ *Edge detection*. Detects a pronounced slope between neighbourhood pixel values, indicating that there is an axis in the ROI.
  ○ *HVLine*. Slope algorithm that generates the possible vertical or horizontal line in the ROI.

Using artificial vision libraries and combining the preprocessing procedures and algorithms with algebraic operations, we implemented an application that was as parameterizable as possible and open to future updates.

**Figure 9.**
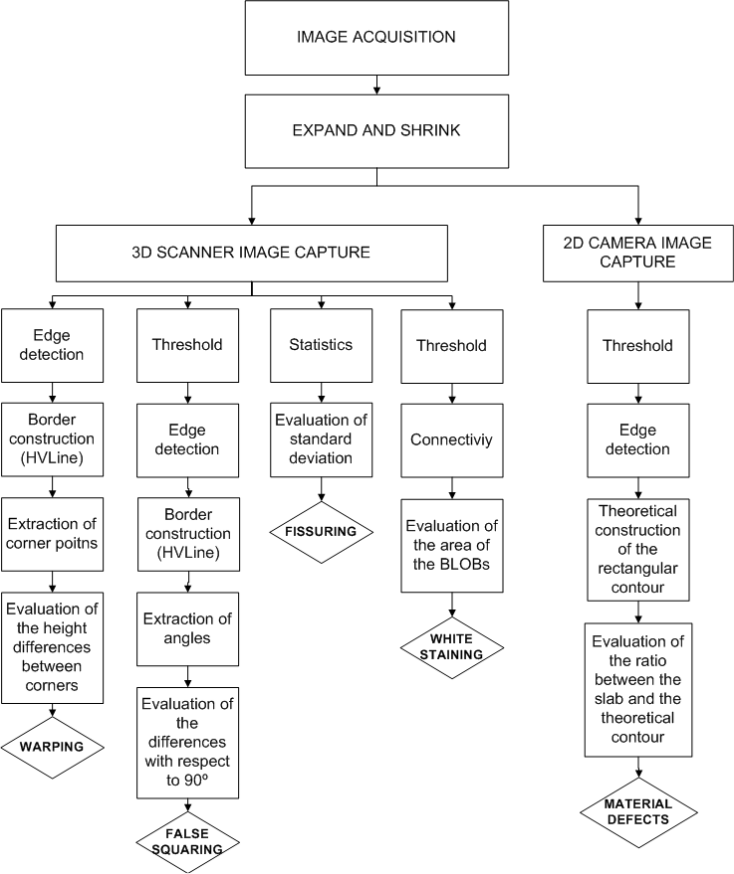
Flow diagram of the algorithms used to detect each kind of defect.

Figure 9 depicts a flow diagram of the algorithms used to detect each kind of defect. Table 2 shows, for our lighting conditions, the threshold values used for each defect if binarization proved necessary for detection.

### 3.2. Results

In order to test the proposed calibration algorithm (4)-(5)-(6), we compared it to the Tsai algorithm (1)-(2)-(3). From Table 3, which shows the results obtained for error (%) with respect to the transversal direction in the 2D case and with respect to slab thickness in the 3D case, it can be concluded that the proposed algorithm is suitable for the studied application, because, although it has errors higher than those of the Tsai algorithm, they remain below the maximums permitted by the standard. In comparing the two methods, the loss of precision is a small tradeoff for the savings in time and complexity.

Table 4 shows the main defects detected by the application and the detection rates.

## 4. Conclusions

We studied the feasibility of automating the quality control and classification process for slate slabs using images produced by a hybrid 3D-2D laser scanning system.

Slate classification to date has typically been performed manually by experts, which implies human subjectivity that can be aggravated by fatigue. In addition, slate experts need very specific and costly training.

The results of our study demonstrate that slate defects can be detected automatically using our hybrid 3D-2D laser scanning system. One important defect, the quartz filament, was excluded but will be studied in future research (in terms of identifying a trend line in the slate, typically a whitish tone). The model was tested in real time as a prototype.

As for the development of the system, we proposed an optimized calibration algorithm based on the Tsai algorithm. Comparing the results obtained for both, the proposed algorithm proved optimal for the application described, as it considerably simplified the calibration process, which is an aspect of crucial importance in an industrial setting.

A further line of research will be to examine the application of machine learning techniques (for example, support vector machines and neural networks) to slate slab classification on the basis of variables representing the information in the slate slabs.

## Figures and Tables

**Table 1.** Defects and limit values established for this application.

**Defect**	**Limit values**
Material defects	98.75%
False squaring	5°
Warping	1.5 mm
Homogeneity/fissuring	Std. Dev. = 8 mm
White staining	1,000 mm^2^

**Table 2.** Defects and threshold binarization.

**Defect**	**Threshold Binarization**
Material defects	5
False squaring	5
Warping	N/A
Homogeneity/fissuring	N/A
White staining	8

**Table 3.** Errors for the different calibration methods and cameras.

**Calibration method-Camera**	**Error (%)**
Tsai method - 2D	0.2
Tsai method - 3D	0.2
Proposed method - 2D	1.5
Proposed method - 3D	1.9

**Table 4.** Defects and detection rates.

**Defect**	**Detection (%)**
Material defects	99.8
False squaring	99.7
Warping	99.5
Homogeneity/fissuring	99.5
White staining	99.8
